# Mathilde Ludendorff (1877–1966): Nervenärztin und völkische Philosophin

**DOI:** 10.1007/s00115-021-01108-x

**Published:** 2021-03-25

**Authors:** Hans Förstl

**Affiliations:** Klinik und Poliklinik für Psychiatrie und Psychotherapie, TU München, Ismaningerstr. 22, 81675 München, Deutschland

**Keywords:** Sigmund Freud, Geschichte der Nervenheilkunde, Emil Kraepelin, Erich Ludendorff, Nationalsozialismus, Albert von Schrenck-Notzing, Georg Stertz, Sigmund Freud, History of neuropsychiatry, Emil Kraepelin, Erich Ludendorff, National Socialism, Albert von Schrenck-Notzing, Georg Stertz

## Abstract

Mathilde Ludendorff (geb. Spiess, verw. von Kemnitz, gesch. Kleine) war eine der ersten Frauen, die im wilhelminischen Deutschland Medizin studierte. Sie schrieb eine feministische Dissertation; gab an, sehr früh Sigmund Freuds Psychoanalyse erfolgreich widerlegt zu haben und zu Emil Kraepelins bester Schülerin avanciert zu sein; deckte den Schwindel von Albert Schrenck-Notzings Mediumforschung auf; firmierte nach 17-monatiger Weiterbildung als Spezialärztin für Nervenheilkunde; behandelte General von Ludendorffs erste Frau und wurde alsbald seine zweite; entwickelte eine germanische Philosophie, die Adolf Hitler zu verwegen erschien; wurde 1949 in einem Spruchkammerverfahren zunächst als Hauptschuldige verurteilt und setzte sich kritisch mit dem Gutachten ihres Kollegen Georg Stertz über ihren eigenen Geisteszustand auseinander. Ihre zahlreichen Schriften werden weiter verlegt. Der von ihr begründete „Bund für Gotterkenntnis (Ludendorff)“ existiert noch und wird vom Verfassungsschutz beobachtet.

## Abstract (en)

Mathilde Ludendorff (nee Spiess, widowed von Kemnitz, divorced Kleine) was one of the first women who studied medicine in Imperial Germany. She wrote a feminist doctoral thesis, refuted Sigmund Freud’s psychoanalysis early in her career, detected the fraud of Albert von Schrenck-Notzing’s spiritualist research, became a specialist for nervous and mental diseases after only 17 months of training with Emil Kraepelin, as his—according to her own words—best pupil, treated General Ludendorff’s first wife and soon became his second, developed a Germanic philosophy too radical for Adolf Hitler’s taste, was considered as a primary culprit after a first denazification trial in 1949 and contested the expert opinion of her colleague Professor Georg Stertz about her own mental state. Her books are still in print and her Alliance for God Cognizance (Ludendorff) still exists and is monitored by the National Intelligence Agency.

Mathilde Friederike Karoline Spiess wurde 1877 als viertes von acht Kindern des früheren Vikars und späteren Gymnasialprofessors Dr. phil. Bernhard Spiess und seiner Frau Johanna in Wiesbaden geboren [4, 40, 47, 49]. Fünf Kinder überlebten und Geld war knapp. Mit 16 Jahren beendete Mathilde die Ausbildung an der höheren Töchterschule und absolvierte 1895 das Lehrerinnenseminar. Nach einer Tätigkeit als Privatlehrerin wechselte sie an ein Mädchenpensionat in Biebrich. Ab 1900 – bis dahin war es für junge Frauen in Deutschland nicht möglich, das Abitur zu absolvieren – studierte sie mit einigem Einsatz und Ehrgeiz am ersten deutschen Mädchengymnasium in Karlsruhe, um in kurzer und damit wirtschaftlich vertretbarer Zeit das Abitur abzulegen, was ihr bereits im Juli 1901 mit nur drei weiteren Schülerinnen gelang. Mit 24 Jahren begann sie im Wintersemester 1901/02 in Freiburg im Breisgau wiederum mit nur drei weiteren Kommilitoninnen das Medizinstudium. Seit einigen Jahren war die Option „weiblicher Ärzte“ in Deutschland diskutiert worden, aber bisher praktizierten nur Ärztinnen, die im Ausland ausgebildet waren [2, 11, 26]. Großen Eindruck machten auf Mathilde die Vorlesungen des berühmten Zoologen August Weismann über die Deszendenztheorie. Das teure Studium finanzierte sie mit eigenem Erspartem, Privatunterricht, Vortragstätigkeit und mit Unterstützung des Allgemeinen Deutschen Frauenvereins. 1902 verlobte sie sich mit dem 4 Jahre jüngeren Gustav Adolf von Kemnitz, einem Kaufmannssohn aus gutem Hause, der nun auch den Plan entwickelte, zu studieren. Nach dem Physikum wechselte sie 1904 als Gasthörerin nach Berlin, wo sich Frauen noch nicht regulär immatrikulieren konnten, und heiratete von Kemnitz. Das junge Ehepaar zog 1905 nach München und beschäftigte sich mit den Ideen der Lebensreformbewegung und mit Lichtbildkunst (dabei entstandene Aktfotografien wurden später von missgünstigen Nationalsozialisten gegen sie verwendet). 1905 kam ihre Tochter Ingeborg^1^ zur Welt, 1909 Zwillinge, die auf die Namen Asko und Hanno hörten.

## Emil Kraepelin und seine Lieblingsschülerin

Im Wintersemester 1910/11 nahm sie das Medizinstudium wieder auf und hörte auch die Psychiatrievorlesung, in der sie bereits entscheidende Anregungen für ihr künftiges Werk erhielt: „Wer das Kolleg des Direktors der Psychiatrischen Klinik, Kraepelin, mit Aufmerksamkeit verfolgte und sich für das Gebiet besonders interessierte wie ich, der wusste ganz genau Bescheid, denn er führte uns viele Fälle des induzierten Irreseins vor, sprach auch über die Angstneurosen durch Höllenvorstellungen und die Entstehung des religiösen Wahnes …“ [29, S. 214]. 1911 legte ihr Ehemann nach dem Studium der Zoologie und Physiologie bereits seine Dissertation vor (summa cum laude). Mathilde von Kemnitz bestand im April 1912 nach dem 10. Semester das Staatsexamen und übernahm bis Anfang 1913 eine Tätigkeit als Medizinalpraktikantin bei Professor Joseph Albert Amann in der II. Gynäkologischen Klinik (Standort Rotkreuz-Krankenhaus). Dort fertigte sie innerhalb von 6 Wochen ihre Dissertation an: *Der asthenische Infantilismus des Weibes in seinen Beziehungen zur Fortpflanzungstätigkeit und geistigen Betätigung* [20]. Darin argumentierte sie, eine „herabgesetzte Gebärtauglichkeit“ der Frauen und eine schlechte Gesundheit der Kinder seien nicht Folge geistiger, schöpferischer Anstrengung, sondern – falls vorhanden – in erster Linie angeboren; das sollte heißen, dass geistige Anstrengung Frauen nicht schadet. Wesentlicher Ansporn für ihre Auffassung war der Protest gegen eine „rassefremde Machtverteilung“ zwischen Mann und Frau, die unter anderem durch die fünf jesuitischen „Ks“ verursacht sei: Kammer, Küche, Kirche, Kinder, Kleider [28, 46, S. 233–235]. Die Arbeit wurde im Mai 1913 erfolgreich verteidigt und danach in Ploetz’ Archiv für Rassen- und Gesellschaftsbiologie veröffentlicht [20]. Im Juli 1913 erhielt sie die Approbation und wirkte danach als Volontärassistentin bei Professor Kraepelin: „Das Praktizieren bei dem berühmten Psychiater Kraepelin hatte mir manch anerkennendes Kopfnicken, ja auch Lob des wortkargen Mannes eingetragen, und er hatte sogar nach der Prüfung im Ärzteexamen gefragt, ob ich mich dem Gebiete der Psychiatrie zuwenden werde“ [25, S. 220]. Kraepelin war dem Einsatz der ersten Ärztinnen – auch seiner eigenen Tochter Toni Kraepelin [39] – gegenüber aufgeschlossen, unter anderem da „vielfach das Erscheinen des Mannes auf der Frauenabteilung stark erregend wirkt (…) Da man anderwärts mit dieser Einrichtung gute Erfahrungen gemacht hat, wird sie sich voraussichtlich auch bei uns einbürgern, sobald einmal brauchbare Kräfte zur Verfügung stehen“ [25]. Nach ihren Angaben war die persönliche Wertschätzung gegenseitig: „Dazu kam die geistige Anregung durch Professor Kraepelin, der es sehr bald recht schätzte, dass ich mich mit den Patienten so eingehend in Beziehung setzte und viel aus ihnen herausbekam“ [29, S. 229]. Allerdings ließ sie auch die weniger erbaulichen Seiten der damaligen Psychiatrie nicht unerwähnt: „gerichtliche Fälle, Zwangsernährung, Lumbalpunktion, nach kurzer stationärer Untersuchung mehrheitliche Weiterverlegung in die Landesirrenanstalt, Träumende, Ruhe, Schlaf, Dauerbad“ [29, S. 229]: „Hier konnte man wenig lernen, es sei denn, bis zu welchen ungeheuerlichen Zuständen das Weiterpflegen unheilbar zerstörter Menschen führt“ [17, S. 230].

## Albert Freiherr von Schrenck-Notzing und die Mediumforschung

**Figure 1:**
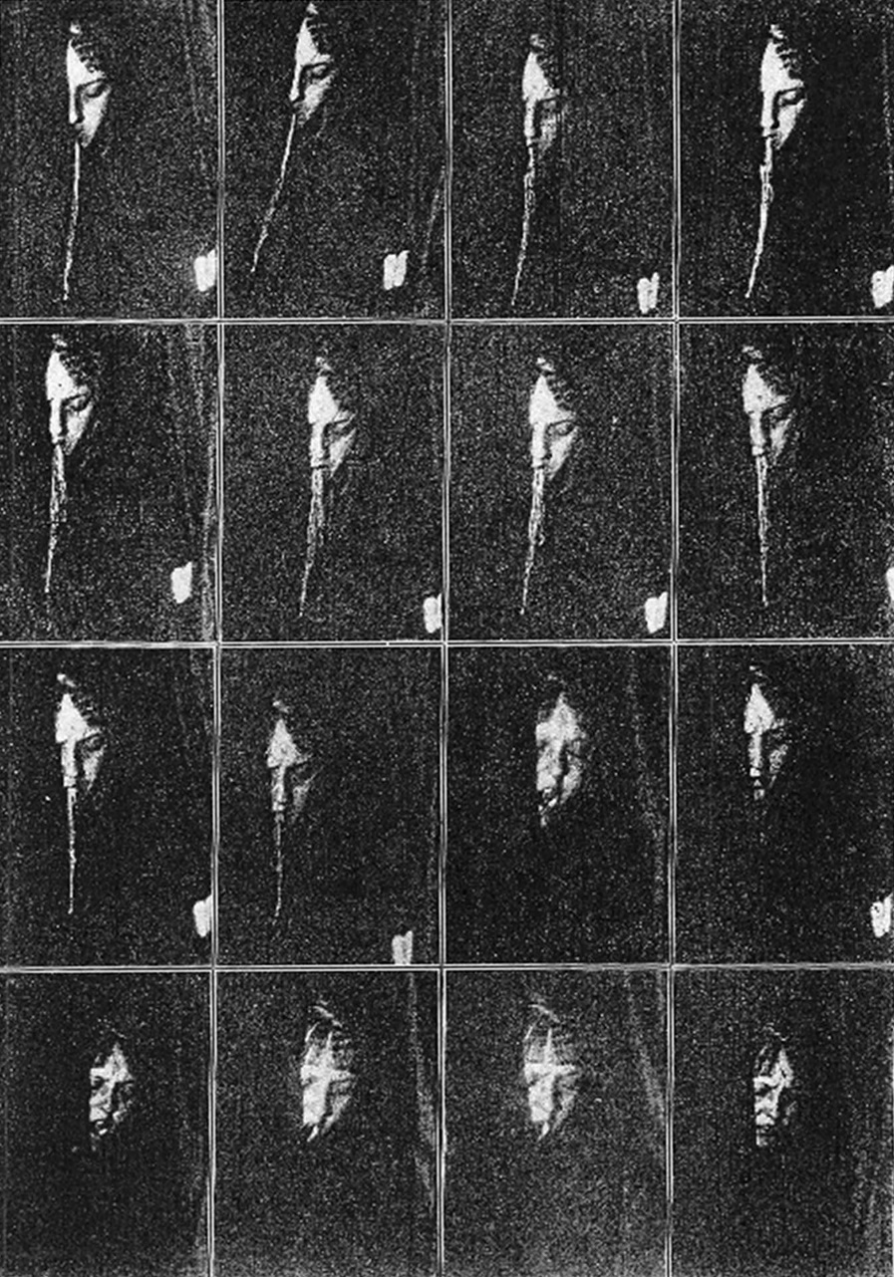

In dieser Zeit gewann sie auch an Ansehen durch ihre kritische Auseinandersetzung mit der parapsychologischen Forschung des Dr. von Schrenck-Notzing, praktischer Arzt. Der lud die Münchner Gesellschaft zu Séancen in sein Palais und suchte Anerkennung in Kreisen der Wissenschaft [8, 45]. Kraepelin betrachtete die obskure Mediumforschung, bei der es sich um meist oral ektoplasmatische Emanationen handelte, mit erheblicher Skepsis. Mathilde von Kemnitz gab Interesse vor und durfte an einer Sitzung teilnehmen. Dabei muss es sich um die Séance am 13.07. 1913 gehandelt haben, für die es recht abweichende schriftliche Berichte aus zwei Quellen gibt – von Schrenck-Notzing [43] und von Kemnitz [21] – sowie erstmals eine kinematographische Aufzeichnung (Abb. 1). Schrenck-Notzing berichtete, das Medium gemeinsam mit einem Kollegen – „Dr. C. (Arzt)“ – vor- und nachkontrolliert zu haben: „In diesen Sitzungen kam das bereits vorbekannte Schleierphänomen aus dem Munde zustande. Öffnung des Vorhanges erfolgte erst, nachdem die Materie produziert worden war“ [43, Ss. 467–468].

Mathilde von Kemnitz hatte vorher schon versucht, das polnische Medium körperlich zu untersuchen was misslang: „Medium: lächelt, schüttelt den Kopf und sagt: ich nicht verstehe“ [21, S. 50 ff.]. Sie beschreibt auch, dass die Untersuchungen seitens der anderen Kollegen keineswegs lege artis durchgeführt werden konnte („sie ist kitzelig“). Zum parapsychologischen Phänomen selbst äußerte sich von Kemnitz höchst abfällig: „um 1 Uhr nachts die langwierige Herrlichkeit des Heraufwürgens einiger Lappen …“ [29, S. 236] und drohte sofort den Schwindel zu entlarven [29, S. 239]. Als Schrenck-Notzing eilends sein großes und reich bebildertes Werk über die Erforschung der mediumistischen Teleplastie veröffentlichte [43], war sie mit einer kritischen Replik schnell zur Stelle [21]. Ihre Arbeit erfuhr sogar eine wohlwollende Besprechung von Eugen Bleuler^2^ [3] und führte zu einer Vortragseinladung auf dem Straßburger Jahreskongress des Deutschen Vereins für Psychiatrie im April 1914, einer Veranstaltung, bei der diesmal die Parapsychologie aufs Korn genommen wurde, nachdem im Jahr zuvor die Psychoanalyse zerlegt worden war [9]. Für Schrenck-Notzing war Mathilde von Kemnitz eine Katastrophe, „imstande den wissenschaftlichen Fortschritt auf Jahre hinaus zu hemmen“ [8, 44, 45]. Für Mathilde von Kemnitz bedeutete der Erfolg eine weitere Motivation für den Kampf gegen obskure Lehren, induziertes Irresein und Verschwörungen aller Art. Sie erweiterte die Front nachdem sie ihren Verstand an Kant, Nietzsche und Schopenhauer geschärft hatte.

## Sigmund Freud und die Seelenkunde

Durchsetzungswille prägte diese Lebensjahre auf ganzer Linie. Gustav Adolf von Kemnitz hatte 1914 seine Habilitationsarbeit vollendet (*Beiträge zur Kenntnis des Spermatozoen-Dimorphismus*), jedoch „unserer Ehe heilige Ausschliesslichkeit hatte nicht gewaltet“ [29, S. 208]. Bei Mathilde wurde eine Lungentuberkulose diagnostiziert und sie begab sich nach Bozen zur Kur, wo sie an ihrem Buch *Das Weib und seine Bestimmung* zu arbeiten begann. Privatdozent Gustav Adolf wurde wegen Kurzsichtigkeit mehrfach für wehruntauglich befunden, die Finanzen waren durch Fehlspekulationen verloren und sie war nach verhältnismäßig kurzer Weiterbildungszeit^3^ gezwungen, eine Anstellung als Spezialärztin für Nervenheilkunde in einem Kur- und Offiziersgenesungsheim in Garmisch anzunehmen, wo sie sich dann beruflich selbständig machte. In dieser Zeit entstand eine Arbeit über traumatische Kriegsneurosen bei Offizieren [22]. Ihr Mann nahm mit seiner jüdischen Geliebten eine Wohnung in Schwabing. Zum Jahreswechsel 1916/17 wurde er auf einer Skitour mit zwei Begleiterinnen von einer Lawine verschüttet, sein dritter Bergunfall in kurzer Folge und diesmal tödlich [38, 47].

Mathilde von Kemnitz hatte nach eigenen Worten „die Freud’sche Theorie … schon in meinen Assistentenjahren widerlegt, ohne dass meine Abhandlung in die Fachpresse aufgenommen worden wäre“ [29, S. 273]. … „Die sogenannte Freudsche ‚ Psychoanalyse‘ hat die Gesetze des Unterbewusstseins zu geringerem Teil begriffen, aber in ihren Ursachen und Zusammenhängen so traurig missverstanden …“ [28, S. 160]. … „Die Lehre der Psychoanalyse Freuds, dies verdrängte Erlebnis sei stets ein sexuelles, ist ein verheerender Irrtum, der sich aus seiner jüdischen Rasseeigenart etwas erklären und entschuldigen lässt“ [28, S. 162]. … „Die Art, wie er (Freud) die Sexualität überhaupt anfasst, die Art wie er sie durch Zotenumdeutungen zu heilen versucht, indem er die ‚freien Assoziationen‘ und die Träume im Sinne von Zoten auslegt, ist ein volksverseuchender Irrsinn. Er wird nur begreiflich, wenn wir sehen, wie völlig die Phantasie dieses chronisch überreizten Juden und seine Heilverfahren mit bestimmten Teilen des Talmuds übereinstimmen. Sein Erbgut im Unterbewusstsein ermöglicht diese Auffassung von der Seele und ihren Gesetzen. … Wer die genannten Gesetze erkannt hat, der wird in der leider eingeführten ungeheuer unbeholfenen und umständlichen, mechanischen Weise ein Unvermögen sehen, das verdrängte Erlebnis durch ‚freie Assoziationen‘ zu finden“ [28, S. 165–166]. Sie selbst habe nur bei 5 % aller Neurosen „sexuelle Erlebnisse“ gefunden. Mit diesen Darlegungen werden Kraepelins Gedanken aus der 8. Auflage seines Lehrbuches drastisch paraphrasiert. Er fand die Auffassungen Freuds höchst eigentümlich: „Jedenfalls genügen die Aussagen einer Reihe auf das stärkste suggestiv beeinflussbarer Personen nicht entfernt, um auch nur die bescheidensten Anforderungen an die wissenschaftliche Begründung derart erstaunlicher Annahmen zu erfüllen“ [24; z. B. S. 1681 ff.]. Wenngleich sich beide ihrer großen Bedeutung bewusst waren, blieben die heuristischen Differenzen zwischen der narrativen Therapie des späteren Literaturnobelpreisträgers und der trockenen Technik des Nosologen zu groß für eine wissenschaftliche oder gar persönliche Annäherung – wobei möglicherweise auch Kraepelins völkische Einstellung eine Rolle spielte [12, 18, 34, 35, 51].

## Erich Ludendorff und die Deutsche Gotterkenntnis

**Figure 2:**
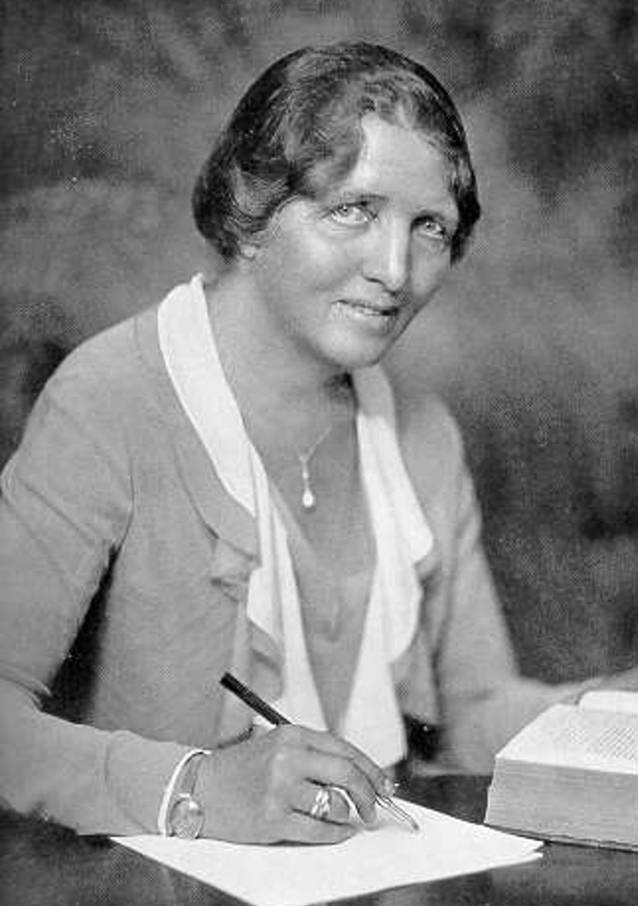

Im Jahr 1919 heiratete Mathilde von Kemnitz Major a. D. Edmund G. Kleine. Die Ehe wurde 1922 geschieden. Bis 1923 hatte sie ihre weltanschaulichen Gedanken in einer Reihe weiterer Schriften dargelegt (Tab. 1; Abb. 2). Im August kam es nach einer Parteikundgebung in der Murnauer Turnhalle zu einer Begegnung mit Adolf Hitler in privatem Rahmen [14, S. 110]: „Frau von Kemnitz widerfuhr bei dieser Gelegenheit das Missgeschick, mit ihrer, von einem durchsichtigen Chiffonkleid eingehüllten imposanten Körpersilhouette derart in das Hintergrundlicht dieser weissen Nacht zu geraten, dass eine stehend von ihr erteilte Vorlesung über Rasseaufzucht und Freikörperkultur gleich eine recht anschauliche plastische Untermalung erfuhr. … An jenem Abend gestaltete sie ihre Auslassungen über Weltgeist und Weltall ein wenig zu ermüdend; schließlich wandte sie sich mit ihrer Forderung nach einer neuen, im nordischen Ahnenerbe wurzelnden Religion direkt an Hitler.“ Hitler reagierte abwehrend, er wolle nicht ausschließen, dass es möglicherweise irgendwann einmal einen Philosophen geben werde, der, aus dem was wir wollen, ein Glaubenssystem entwickeln werde. „Darauf Frau Mathilde, sich zu voller Körpergrösse straffend und das Denkerhaupt empor gereckt als lausche sie bereits dem Flügelschlag der Ewigkeit: Dieser Philosoph, Herr Hitler, steht bereits vor Ihnen!“ Er verabschiedete sich daraufhin umgehend und wortlos [14, S. 111]. Noch 1942 äußerte sich Hitler im Tischgespräch unvorteilhaft über früher gewonnene Eindrücke [17]: „1924 tauchten bei mir die politischen Weiber auf: die Frau [Ehrengard] von Treuenfels, die Mathilde von Kemnitz, sie wollten Reichstagsmitglieder werden, um die Sitten dort zu veredeln. Ich sagte Ihnen, neunundneunzig Prozent aller Beratungsgegenstände sind Männerdinge, die sie nicht beurteilen können!“

**Table 1:** 

1913	Der asthenische Infantilismus des Weibes in seinen Beziehungen zur Fortpflanzungstätigkeit und geistigen Betätigung (Med. Diss.)
1914	Ein Blick in die Dunkelkammer der Geisterseher: moderne Medium-„Forschung“: kritische Betrachtung zu Dr. von Schrenck-Notzings „Materialisationsphaenomene“
1917	Das Weib und seine Bestimmung. Ein Beitrag zur Psychologie der Frau und zur Neuorientierung ihrer Pflichten
1919	Erotische Wiedergeburt (später: Der Minne Genesung)
1920	Des Weibes Kulturtat
1921	Triumph des Unsterblichkeitswillens
1923	Der Seele Ursprung und Wesen (3 Bände) I. Schöpfungsgeschichte
1925	II. Des Menschen Seele
1927	III. Selbstschöpfung
1927	Deutscher Gottglaube
1929	Das Geheimnis der Jesuitenmacht und ihr Ende (mit E. Ludendorff)
1930	Der Seele Wirken und Gestalten I. des Kindes Seele und der Eltern Amt
1933	II. die Volksseele und ihre Machtgestalter
1935	III. das Gottlied der Völker – eine Philosophie der Kultur
1933	Induziertes Irresein durch Occultlehren
1934	Christliche Grausamkeit an Deutschen Frauen (mit Walter Löhde)
1936–68	Statt Heiligenschein oder Hexenzeichen – mein Leben, 6 Bände
1939	Die Judenmacht, ihr Wesen und Ende
1941	Der Siegeszug der Physik – ein Triumph der Gotterkenntnis meiner Werke
1950	Wunder der Biologie im Lichte der Gotterkenntnis meiner Werke
1957	Das Hohe Lied der göttlichen Wahlkraft
1959	In den Gefilden der Gottoffenbarung
1960	Das Jenseitsgut der Menschenseele I. Der Mensch, das grosse Wagnis der Schöpfung
1961	Unnahbarkeit des Vollendeten
1962	Von der Herrlichkeit des Schöpfungszieles

Ab 1925 behandelte sie die morphiumsüchtige Ehefrau Erich Ludendorffs, Margarethe, von der er sich dann im Juli 1926 scheiden ließ, um im September des gleichen Jahres Mathilde auf dem Standesamt in Tutzing zu ehelichen. Dennoch fand die geschiedene Margarethe in ihren etwas konfusen Erinnerungen („bei dem Hitler-Putsch passierte eine amüsante Geschichte …“; [32, S. 309]) fast nur gute Worte für ihren geschiedenen Gatten, außer „Menschenkenntnis hat Ludendorff nie besessen, sonst könnte er nicht immer Einflüssen unterliegen, die ihn ins Unglück stürzen“ [32, S. 276]. Mathilde Ludendorff schildert im Kapitel *Der Menschen Niedertracht öffnet dem Glück die Tore *im Band 5 ihrer Autobiographie *Freiheitskampf wider eine Welt von Feinden* [31] wie sie versuchte, mit ihrem ärztlichen Gewissen ins Reine zu kommen: „Wie schwer und unerquicklich ist doch der Arztberuf. Hätte die Patientin die Widerstandskraft bewahrt, dann war ich der Engel, der sie gerettet hatte. Nun trafen mich mit einem Mal Hass und Verleumdung. Gut, dass Ludendorff das nicht ahnte“ [32, S. 47]. Es werde behauptet „eine Ärztin, Frau Dr. v. Kemnitz, habe unter dem Deckmantel ärztlicher Hilfe die Ehe unterwühlt, und Ludendorff sei ihrer Taktik erlegen. So etwa lauteten diese Mitteilungen, die in geringen Abwandlungen in den verschiedensten Zeitungen in ganz Deutschland auftauchten und meine Frauen- und Berufsehre besudeln sollten“ [32, S. 49]. *… *„Und sieh da, der Tag war gekommen, der mich in eine Welt des Glückes stellte, eine so völlig andere Welt der seelischen Erfüllung in Zweisamkeit. Es hatte der einsame, für alles seelisch Unnahbare, Verschlossene, Grosse, der sich ein Leben lang – oft sogar von seiner nächsten Umgebung – lieber missverstehen liess, als Unebenbürtigen eine Wirkung auf sein Seeleninneres zu gewähren, seine Seele aufgetan“ [32, S. 53]. Mit Erich Ludendorff (1865 bis 1937) heiratete sie die Personifikation des deutschen Kriegshelden und seiner Schmach, den deutschen De-facto-Strategen des 1. Weltkriegs, der 9 Mio. tote Soldaten mitzuverantworten hatte, sich zur Ehrenrettung der Dolchstoßlegende bediente, gerade bei der Wahl zum Reichspräsidenten mit nur 300.000 Stimmen abgestraft worden war und später unter Mathildes Einfluss den totalen Krieg als geballte Anstrengung der „seelischen Entschlossenheit“ einer ganzen Nation ersann [27].

**Figure 3:**
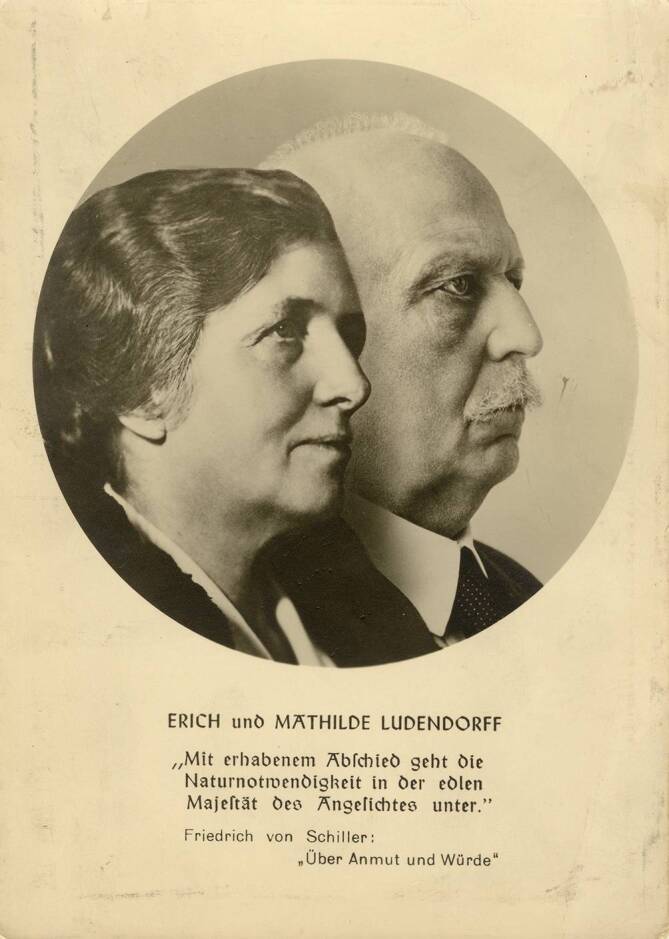

Hier begegneten sich verwandte Seelen: „Das nordische Gotterleben, also vor allem gekennzeichnet durch das klar bewusste Erleben des Gottes in der eigenen Seele, vererbt hiermit die starke Entfaltung des Gottesstolzes im Rassecharakter. Daher der Freiheitswille und das Herrenbewusstsein des Germanen“ [30, S. 137]. Ludendorffs kongeniale Einschätzung seiner Gattin [7]: „Sie zeigte Wirken der Volksseele – das heisst des Rasseerbgutes im Unterbewusstsein – wie es Gott erlebt und dem Göttlichen gegenübersteht mit den Charaktereigenschaften der Rasse – in den einzelnen Volkskindern und wie sie in rassereinen Völkern, aber selbst noch in rassegemischten, deren Hüterin werden kann.“ Zwei verwandte Seelen hatten sich gefunden, gründeten 1928/29 *Ludendorffs Volkswarte Verlag*, 1937 – mit zögerlicher Erlaubnis Hitlers, der Ludendorff wieder für sich gewinnen wollte – den völkisch-religiösen *Bund für Deutsche Gotterkenntnis (L)* [L für Ludendorff] und feierten einander in hohem Ton (Abb. 3), manövrierten sich damit jedoch in eine immer extremere Außenseiterposition. Goebbels [13]: „Der Chef spricht sich scharf gegen Ludendorff, vor allem gegen seine Frau aus“ (29.05.1929). „Es ist schade um den grossen General. Seine Frau ist ein böser Geist“ (23.05.1937). Erich Ludendorff starb im Dezember 1937 in München. Zu Kriegsbeginn wurde dem Ludendorff-Verlag das zugestandene Papierkontingent prompt entzogen, sodass auch die Halbmonatsschrift *Am Heiligen Quell Deutscher Kraft* versiegte. Mathilde und Erich Ludendorff hatten sich in ihre eigene ultravölkische Lehre hineingesteigert und den Nationalsozialismus Hitlers sichtlich geringgeschätzt. Sie waren keine Mitglieder der NSDAP oder anderer nationalsozialistischer Organisationen. Dennoch stand Mathilde im Verdacht, intellektuelle (Mit‑)Urheberin des NS-Gewaltstaates zu sein, und musste sich – wie sehr viele andere – im Rahmen der Entnazifizierung einem Spruchkammerverfahren stellen [6, 47].

## Georg Stertz und das Spruchkammerverfahren

Georg Stertz (1878–1959) war 1937 wegen seiner Ehe mit der „halbjüdischen“ Tochter Aloys Alzheimers in Kiel als Direktor der Psychiatrischen Universitätsklinik zwangsemeritiert worden und gelangte 1947 zu der späten Ehre, Bumkes Nachfolger an der Münchner Nervenklinik zu werden. Im gleichen Jahr ereilte ihn der Auftrag von der Spruchkammer des Landkreises Starnberg, ein Gutachten über den Geisteszustand Mathilde Ludendorffs zu verfassen. Einer Bekannten Mathilde Ludendorffs gegenüber äußerte Stertz im Mai 1947, „dass es besser wäre, man würde Frau Dr. Ludendorff als nicht ganz geistig verantwortlich für ihre Werke erklären. Dann wäre das Verfahren schnell beendet. Diese Frau Dr. von Unruh wies dies Ansinnen mit der Erwiderung zurück … dass sie völlig gesund sei und von Prof. Kraepelin seinerzeit als seine beste Schülerin bezeichnet worden sei *…*“ [50].

Das Gutachten mit Datum vom 22.09.1947 [48] nimmt zu den Fragen Stellung, ob Mathilde Ludendorff für ihr Tun und für ihre Werke in Vergangenheit und Gegenwart die volle Verantwortung besitze (damals § 51 StGB) und ob sie einweisungsfähig für ein Arbeitslager sei. Die zweite Frage wollte Stertz angesichts der „annähernd 50-jährigen“ (sie war damals fast 70) Patientin nicht beantworten. Auf S. zwei steht „nach Bekundungen einiger verlässlicher Zeugen soll (Mathilde Ludendorff) … zu Beginn ihrer ärztlichen Tätigkeit einen auffälligen, extravaganten Eindruck gemacht haben, der sich in ihrem Äusseren und in ihrem Benehmen zu erkennen gab.“ Kurz erwähnt werden Studium und ärztliche Tätigkeit sowie die Hinwendung zur schriftstellerischen Tätigkeit nach der Lektüre Kants und Schopenhauers mit dem propagandistischen Ziel einer Bekämpfung überstaatlicher Mächte. Ab der fünften Seite mischen sich Stertz’ grundsätzliche psychopathologische Überlegungen und diagnostische Einschätzungen über das Ehepaar Ludendorff, insbesondere hinsichtlich der vollkommenen Arbeitsgemeinschaft und der Grenzen von Selbstüberschätzung und Größenwahn. Auf S. 16 gelangt Stertz zu dem Schluss das „gesamte Verhalten von Frau Dr. Ludendorff, ihre Intelligenz, ihre lebhafte jeder Anregung zugewandte Auffassungsgabe, ihr Temperament, ihr ungestörtes Gedächtnis, das alles spreche gegen das Bestehen einer geistigen Erkrankung. …“ Es gebe keinerlei Anhaltspunkte dafür, dass sie nicht voll verantwortlich wäre.

Mathilde Ludendorff, Facharzt für Psychiatrie [30], ließ dem Gericht eine 14-seitige Kritik des Gutachtens zukommen, in dem sie unter anderem die lange Latenz in der Gutachtenerstellung monierte, falsche Angaben hinsichtlich Zeit der Assistententätigkeit bei Kraepelin (nicht 1 Jahr, sondern 1,5, nämlich vom 01.08.1912 bis 01.01.1914). Das Gutachten sei vom Standpunkte des Facharztes aus „geradezu ungeheuerlich“, u. a. da es sich der Werke des Pamphletisten Martini [33] bediene^4^. Zur Selbstüberschätzung könne Stertz kein Urteil abgeben, da er keine Zeit gehabt habe, ihre Leistung, ihre Werke zu würdigen. Stertz’ „psychiatrisches Kolleg“ über Wahn gehöre nicht in das Gutachten. „Er selbst hat sein Liedlein von den armen Juden gesungen.“ Nach ihrem Eindruck schien er keine genauen Aufzeichnungen über die Unterredungen gemacht zu haben, daher die Erinnerungsirrtümer.

Mathilde Ludendorff dagegen hatte selbst ein detailliertes 31-seitiges, teilweise wörtliches Gedächtnisprotokoll über die insgesamt fünf Begegnungen angefertigt [30]. Ende Mai habe er sie gefragt, ob sie okkult-gläubig sei, also gar nichts über ihre Arbeit gewusst. Anfang Juni sei er auf die psychische Erkrankung ihrer älteren Schwester zu sprechen gekommen um dann anzumerken „und dann haben Sie sich auf einmal … zugetraut, in das schwierige Problem der Atomphysik einzudringen? (diese Frage ist von besonders ironischem Lächeln begleitet).“ Mitte Juni habe er sich über die dichterische Qualität ihres Werkes mokiert: „er bricht in Lachen aus und kann zunächst gar nicht damit aufhören, unterbricht auch im Folgenden immer wieder sein Sprechen mit höhnischem Lächeln oder Lachen. Sie haben behauptet, dass die Freimaurer Schiller, Lessing, Mozart und andere ermordet hätten und das nenne ich Wahnsinn.“ Am 26.07.1947 um 16:30 findet die letzte Begegnung statt: „Professor Stertz empfängt mich in seinem Schlafzimmer, sitzt in Pyjama im Sessel, spricht nur sehr leise und matt. Wir bedauern gegenseitig das durchlebte Kranksein. … Die Frau Professor kommt und sagt, ihr Mann müsse nun ins Bett, die Stunde sei vorüber. Frau Professor will mir die Bücher zurückgeben, führt mich von der Privatwohnung durch die Männerabteilung der Klinik und führt mich in ein Zimmer, an dessen Tür das Schild: ‚Dr. Röhrig‘ hängt. Dort liegen auf dem Schreibtisch alle meine Bücher, die sie mir mitgibt.“

## Epilog

Mathilde Ludendorff wurde im ersten Spruchkammerverfahren als Hauptschuldige verurteilt. 1951 wurde das Urteil revidiert und abgemildert und sie wurde als „Belastete“ eingestuft, die den Nationalsozialismus als Aktivistin unterstützt habe. Hauptschuldigen (Kategorie I) drohten ursprünglich 2 bis 10 Jahre, Belasteten (Kategorie II) bis zu 5 Jahre Arbeitslager mit nachfolgendem Arbeitsverbot und Enteignung. Auch Minderbelastete (III) und Mitläufer (IV) standen in der Gefahr, Arbeit, Ansprüche auf Lebensmittelmarken und Rente zu verlieren. Dabei gab es allerdings zwischen und auch innerhalb der Besatzungszonen keine einheitlichen Richtlinien, jedoch eine deutliche Abmilderung der Strafmaße innerhalb ganz weniger Jahre; nur 0,7 % der Beschuldigten wurden als Hauptschuldige oder Belastete eingestuft [5, 6, 36]. 1951 wurde der völkische „Bund für Gotterkenntnis“ wieder ins Leben gerufen und 1961 als verfassungsfeindlich verboten. 1971 wurde der Bund wegen Verfahrensfehlern wieder zugelassen. Mathilde Ludendorff starb 1966 im Alter von 88 Jahren in Tutzing an einer Urämie [4, 40, 47, 49]. Die Bücher von Mathilde Ludendorff werden weiterhin verlegt (*hohewarte-online.de*). Der Verlag versucht sich auch lebhaft in aktuelle Debatten einzubringen. Die Ludendorff-Villa in Tutzing steht seit 2010 unter Denkmalschutz und den Verein für Gotterkenntnis (L) gibt es immer noch: „Wenn Sie Verbindung mit uns aufnehmen wollen, schreiben Sie uns an folgende Anschrift oder schicken Sie uns eine E‑Post (E-Mail) über unser Kontaktformular: Weltanschauungsgemeinschaft Bund für Gotterkenntnis (Ludendorff) e. V., Postfach 1254, 82324 Tutzing/Oberbayern. E‑Post: info(AT)ludendorff.info.“

Es gibt keine zuverlässigen Zahlen, wie viele dem Bund für Gotterkenntnis noch anhängen, aber während anhaltender Krisen und in Phasen der Orientierungslosigkeit nimmt das Interesse an Querdenken und alternativen Erklärungsmodellen der Realität zu. Sie bieten angesichts unübersichtlicher Verhältnisse schlüssige (konklusive), dank höheren Wissens exklusive und wegen der Gruppenzugehörigkeit inklusive Alternativen [10]. Während der Weimarer Republik erstarkten neu-heidnische Glaubensbewegungen, die alte Kulte reaktivierten, z. B. Wilhelm Hauers Deutsche Glaubensbewegung, Sigrid Hankes Unitarier und eben auch Ludendorffs Deutscher Gottglaube [23, 37, 47, 49]. Ein anderer Kraepelin-Schüler, Willi Hellpach, der 1925 deutlich erfolgreicher als Ludendorff für das Amt des deutschen Reichspräsidenten kandidiert hatte, profilierte sich auch als Pantheist, wirkte aber gleichzeitig als Realpolitiker, begründete die Umweltpsychologie und beförderte die Psychosomatik [15, 16]. Faszination und Faschismus hängen zusammen. Der Schwung des Neo-Paganismus wurde von den Ideologen Feser und Rosenberg für die Hitler-Bewegung aufgenommen – wobei sich Ludendorffs partout nicht fügen wollten [14, 23, 37, 49]. Hitlers gewählt ekstatische Rhetorik und seine inbrünstigen Posen schöpften aus dieser quasi-religiösen Quelle.

Das Wesen von Mathilde Ludendorffs schillernden Werken lässt sich nicht einfach ergründen. Es reicht von antiokkultistisch bis esoterisch, feministisch bis pantheistisch, antiklerikal bis antisemitisch, völkisch bis verschwörungstheoretisch und verdient das Interesse von Nervenärzten – auch hinsichtlich der Begutachtung narzisstischer und querulatorischer Personen mit Fachkenntnissen.

## Footnotes

1 Mutter des Chemikers Walter E. Karg von Bebenburg (1927–1980), Inhaber mehrerer Patente, u. a. für das Schmerzmittel Flupirtin (Katadolon^(R)^); als Künstler: Walter E. Richartz, Übersetzer und von der Literaturkritik hochgelobter Autor [1], z. B. von „Tod den Ärtzten“ [41], das u. a. eine ausführliche und ironische Auseinandersetzung mit Verschwörungstheorien enthält. Der Enkel spielt in den Erziehungswerken seiner Großmutter eine bedeutende Rolle.

2 Dies ist nicht nur als Zeichen der Anerkennung in der Fachwelt erwähnenswert, sondern vor allem wegen Bleulers eigenem Interesse am Okkultismus [8].

3 Richtlinien zu Inhalt und Dauer der Facharztweiterbildungen wurden erst 1924 beschlossen [19].

4 Der Journalist Winfried Martini war von Staatsanwalt Julius Herf ebenfalls zu einer Stellungnahme aufgefordert worden und hatte bei Ludendorffs die Diagnose eines „germanisch-depressiven Irreseins“ gestellt [5, 33].
