# Erratum zu: Mathilde Ludendorff (1877–1966): Nervenärztin und völkische Philosophin

**DOI:** 10.1007/s00115-021-01228-4

**Published:** 2022-01-25

**Authors:** Hans Förstl

**Affiliations:** Klinik und Poliklinik für Psychiatrie und Psychotherapie, TU München, Ismaningerstr. 22, 81675 München, Deutschland

**Erratum zu:**

**Der Nervenarzt 2021**

10.1007/s00115-021-01108-x

Der Beitrag enthält eine fehlerhafte Information. Bitte beachten Sie: Sigmund Freud (1856–1939) hat auch den Nobelpreis für Literatur nicht erhalten.

Please note: Sigmund Freud (1856–1939) did not even receive the Nobel Prize for Literature.

